# Supplementary material for: Optimizing hypertension prediction using ensemble learning approaches
Source: PLoS One. 2024 Dec 23;19(12):e0315865. doi: 10.1371/journal.pone.0315865 (PMC11666061; doi:10.1371/journal.pone.0315865)
Supplement: S1 Appendix — (DOCX) [file pone.0315865.s001.docx]

**Machine Learning Algorithms:**

**Logistic Regression (LR)**

One of the most often used supervised machine learning algorithms that make use of probability is logistic regression (LR). The most well-liked supervised machine learning algorithm, LR, is mostly applied to classification tasks[1]. This model utilizes the logistic function to predict the likelihood of the response (HTN and non-HTN) based on various input features. The logistic function can be represented as follows

$Logit\left( p_{j} \right)=log\left( \frac{p_{j}}{1-p_{j}} \right)=\beta_{0}+\beta_{1}x_{1j}+\beta_{2}x_{2j}+...+\beta_{k}x_{kj}+\epsilon_{j}, j=1,2,...,n\text{ }$

Where,$p_{j}$denote the probability of HTN for *j^th^* individual;$X_{kj}$is the *k^th^* input feature of the *j^th^* individual and$\beta_{k}$is the *k^th^* regression coefficients.

The above equation (i) can be expressed as

$P=\frac{exp\left( \beta_{0}+\beta_{1}+\beta_{2}+...+\beta_{k}x_{kj} \right)}{1+exp\left( \beta_{0}+\beta_{1}+\beta_{2}+...+\beta_{k}x_{kj} \right)}\text{ }$

and odds as

$\frac{p}{1-p}=exp\left( \beta_{0}+\beta_{1}+\beta_{2}+...+\beta_{k}x_{kj} \right)\text{ }$

If $\frac{p}{1-p}>1,$then we classify as HTN, while $\frac{p}{1-p}<1,$then we classify as non-HTN.

**Artificial Neural Network (ANN)**

Artificial Neural Network (ANN) attempts to mimic the human brain to learn complex tasks. It is modeled as interconnected group of nodes in a way which is like the vast network of neurons in the human brain[2]. It is made up of interconnected processing nodes arranged into three categories: hidden, output, and input layers. The hidden layer is connected to the output, while the input layer is connected to the hidden layer with the updated weight. This method maps the link between input characteristics and result variable by using *X = x_1_, x_2_,...,x_k_* as the input vector in the back propagation (BP) algorithm for learning. The BP approach uses a non-linear sigmoid activation function to modify the weights of hidden layers in a backward direction, propagating the error between the input risk factors and result variable[3]. The sigmoid activate function is defined as

$Sigmoid\left( x \right)=\frac{1}{1+e^{-x}}$

**Random Forest (RF)**

RF is very efficient in both classification and regression. It uses the DT algorithm as the base classifier with a bagging approach to generate multiple small decision trees by random sampling with replacement [4]. Each decision tree is built based on various sub-datasets and features. This approach allows reducing variance in decision trees [5]. Steps involved in RF Algorithm

- **Step 1:** In the Random Forest model, a subset of data points and a subset of features is selected for constructing each decision tree. Simply put, n random records and m features are taken from the data set having k number of records.
- **Step 2:** Individual decision trees are constructed for each sample.
- **Step 3:** Each decision tree will generate an output.
- **Step 4:** Final output is considered based on majority voting or averaging for classification and regression, respectively.

**Extreme gradient boosting (XGB)**

XGBoost is designed to be highly efficient, flexible, and portable to solve both classification and regression problems[6]. Boosting is a learning algorithm, which attempts to create a strong classifier based on weak learners or classifiers. The weak and strong classification models mention to the correlation of predicted and actual class. By adding classifiers on top of each other iteratively, the next classifier can modify the errors of the earlier one. This procedure is repeated until the training data set accurately predicts the membership class label of the target variable[7]. Mathematically the predictions from the trees can be expressed as:

$$\hat{y}=\phi\left( x \right)=\frac{1}{n}\sum_{k=1}^{n} f_{k}\left( x \right)$$

where 𝑌̂ is the predicted 𝐸𝑇𝑜 , 1 ≤ 𝑘 ≤ 𝑛, and 𝑛 is the total number of functions learnt by the 𝑛 number of trees. The following regularized objective L(𝜙) is minimized to learn the set of functions 𝑓_k_ used in the model:

$$L\left( \phi\right)=\sum_{i} l\left( \hat{y_{i}},y_{i} \right)+\sum_{k} \Omega\left( f_{K} \right)$$

Where $\Omega\left( f_{k} \right)=\gamma T+1/2\lambda\|w{\|}^{2}$

where 𝑙 is a differentiable convex loss function that measures the difference between ̂𝑦_i_ (prediction) and 𝑦_i_ (target). 𝛺 is an extra regularization term that penalizes the growing of more trees in the model to prevent complexity and thus, reduce overfitting. 𝛾 is the complexity of each leaf, 𝑇 is the number of leaves in a tree, 𝜆 is a penalty parameter, and ‖𝑤‖ is the vector of scores on the leaves [8].

**Light Gradient Boosting Machine (LGBM)**

The state-of-the-art LGBM algorithm has been used to predict hypertension. LGBM is a gradient boosting framework that uses tree-based learning algorithms. It is designed to be distributed and efficient using two novel techniques: Gradient-based One-Side Sampling (GOSS) and Exclusive Feature Bundling (EFB)[9]. LGBM trains the multiple tree models in an additive manner, with each new tree model being trained to predict the residuals (i.e., errors) of the prior models[10]. Its main difference from the XGBoost model is that it uses histogram-based algorithms to speed up the training process, reduce memory consumption and employ a leaf-wise growth strategy with depth constraints. The basic idea of histogram algorithm is to discretize continuous floating-point eigenvalues into 𝑘 bins and construct a histogram with a width of 𝑘 [11]. LGBM algorithm is based on decision tress therefore the formulation of the model is as follows. Given a training data set $S=\left( x_{i},y_{i} \right);i=1,2,\cdot\cdot\cdot,n;x_{i}\in R^{m},y_{i}\in R$ where *n* is the sample with *m* features. To find the estimation, the decision tress predictions are combined as follows;

$$\hat{y}_{i}^{LG}=\sum_{p=1}^{p} f_{p}\left( xi \right)$$

where the number of trees is *p* with *f_p_* as trees. The goal is to minimize the objective function below to obtain *f_p_.*

$$f_{p}=arg\min_{f_{p}}\sum_{i=1}^{1} L\left( y_{i},{ŷ}_{i}^{LG\left( p \right)} \right)+\Omega\left( f_{p} \right)$$

The loss function is *L* and the regularization parameter **Ω** which is given by

$$\Omega\left( f_{p} \right)=\alpha T+\frac{1}{2}\lambda\sum_{j=1}^{T} w_{j}^{2}$$

Where *α* and *λ* are the penalty parameters for *T* leaves and weight of leaves *w*. Taking *L* as a loss function which is a squared error, then

${L\left( y_{i},\hat{y}_{i}^{LG\left( p-1 \right)}+f_{p}\left( x \right) \right)=\left( y_{i}-\hat{y}_{i}^{LG\left( p-1 \right)}-f_{p}\left( x \right) \right)}^{2}\left( r-f_{p}\left( x \right) \right)^{2}$

the residual *r* is fitted to obtain *f_p_*. The function for minimizing the objective function at iteration *p* is defined using a quadratic approximation as

$f_{p}\simeq arg{\min_{f}}_{p}\sum_{i=1}^{n} \left[ g_{i} | f_{p}\left( x_{i} \right)+1/2h_{i}f_{i}^{2}\left( x_{i} \right) \right]+\Omega\left( f_{p} \right)$,

$g_{i}=\partial_{\hat{y}_{i}^{LG\left( p-1 \right)}}L\left( y_{i},{ŷ}_{i}^{LG\left( p-1 \right)} \right)$ ,

$h_{i}=\partial_{\hat{y}_{i}^{LG\left( p-1 \right)}}^{2}L\left( y_{i},{ŷ}_{i}^{LG\left( p-1 \right)} \right)$,

Through minimizing the objective function, a new tree *f_p_* is obtained. Each mode with the biggest information gain is divided by the decision tree. The variance gain for a node that separates feature *j* at point s is given by

$$Z_{j\mid0}(s)=\frac{1}{n_{0}}\left\{ \frac{\left( \sum_{\left\{ x\in\in O:x_{i}\leq s \right\}} g_{i} \right)^{2}}{n_{l|0|}^{j}(s)}+\frac{\left( \sum_{\left\{ x_{i}\in O:x_{j}>s \right.} g_{i} \right)^{2}}{n_{r|0|}^{j}(s)} \right\},$$

O is samples on the decision tree fixed node $n_{o}=\sum I\left[ x_{i}\in O \right]$,

$n_{l/o}^{j}\left( s \right)=\sum I\left[ x_{i}\in O:x_{ij}\leq s \right]$ and $n_{l/o}^{j}\left( s \right)=\sum I\left[ x_{i}\in O:x_{ij}\leq s \right]$. The decision tree selects$s_{j}*arg\max_{s}z_{j}\left( s \right)$ for each feature *j* and computes the highest gain Z_i_(s_j_^*^). The data will be split into the right and left nodes according to feature *j** at point *s_j_^*^*. All samples are scanned to find the optimal splitting point in order to calculate the information gain.

**Implementation of the Proposed Stacking Model**

The stacking technique is a meta-learning approach that builds its model using the predictions of multiple base learners as input to train a new meta learner, and then make the final prediction [12]. This can be especially helpful in noisy datasets, because the stacked ensemble can reduce the impact of noisy data by using diverse models, optimizing the combination of base models, and weighing the models based on performance in different ranges of the prediction[13–15]. The algorithm explains how our proposed Stacking model yielded such results. Five base classifiers, namely LGBM, LR, ANN, RF, and XGB, are initialized at level 0 and trained on the training data. Every classifier learns a different pattern and makes predictions. The final predictions are made by the meta layer classifiers using the classifications produced by the base machine learning machine classifiers.

**Algorithm:**

1. Create base layer classifiers
2. LR = (level 0)
3. ANN = (level 0)
4. RF = (level 0)
5. XGB = (level 0)
6. LGBM = (level 0)
7. **For** t=1 to T **do**
8. Learn h_t_ based on D
9. **End For**
10. Construct a new dataset of predictions
11. **For** i = 1 to m **Do**
12. Dh = {x′_i_,y_i_}, where x′_i_ = {h_1_(x_i_),...,h_t_(x_i_)}
13. **End For**
14. Create meta layer classifier
15. Logistic Regression = (level 1)
16. learn H based on D_h_
17. return H
18. Train the model
19. Fit the model (x_train, y_train)
20. Final Prediction
21. End

Each base model is trained on the dataset D over several iterations (for t=1 to T) to learn the prediction function h_t._ After training, a new dataset is constructed using the predictions from the base models. The trained stacked model is used to make the final predictions.

**Reference:**

1. Priya Ranganathan; C. S. Pramesh; Rakesh Aggarwal Common Pitfalls in Statistical Analysis: Logistic Regression. *Perspect. Clin. Res.* **2017**, 148–151.

2. *The Handbook of Brain Theory and Neural Networks*; 2003; Vol. 41;.

3. Montesinos López, O.A.; Montesinos López, A.; Crossa, J. Fundamentals of Artificial Neural Networks and Deep Learning BT - Multivariate Statistical Machine Learning Methods for Genomic Prediction. **2022**, 379–425.

4. Andy, L.; Matthew, W. Classification and Regression by RandomForest. *R News* **2002**, *2*, 18–22.

5. Dinh, A.; Miertschin, S.; Young, A.; Mohanty, S.D. A Data-Driven Approach to Predicting Diabetes and Cardiovascular Disease with Machine Learning. *BMC Med. Inform. Decis. Mak.* **2019**, *19*, doi:10.1186/s12911-019-0918-5.

6. Chen, T.; Guestrin, C. XGBoost: A Scalable Tree Boosting System. *Proc. ACM SIGKDD Int. Conf. Knowl. Discov. Data Min.* **2016**, *13*-*17*-*Augu*, 785–794, doi:10.1145/2939672.2939785.

7. Islam, M.M.; Alam, M.J.; Maniruzzaman, M.; Ahmed, N.A.M.F.; Ali, M.S.; Rahman, M.J.; Roy, D.C. Predicting the Risk of Hypertension Using Machine Learning Algorithms: A Cross Sectional Study in Ethiopia. *PLoS One* **2023**, *18*, doi:10.1371/journal.pone.0289613.

8. C. CHEN, T.. G. XGBoost: A Scalable Tree Boosting System. Proceedings of the 22nd ACM SIGKDD International Conference on Knowledge Discovery and Data Mining. *San Fr. Calif.* **2016**.

9. Ke, G.; Meng, Q.; Finley, T.; Wang, T.; Chen, W.; Ma, W.; Ye, Q.; Liu, T.Y. LightGBM: A Highly Efficient Gradient Boosting Decision Tree. *Adv. Neural Inf. Process. Syst.* **2017**, *2017*-*Decem*, 3147–3155.

10. Chen, T.; Xu, J.; Ying, H.; Chen, X.; Feng, R.; Fang, X.; Gao, H.; Wu, J. Prediction of Extubation Failure for Intensive Care Unit Patients Using Light Gradient Boosting Machine. *IEEE Access* **2019**, *7*, 150960–150968, doi:10.1109/ACCESS.2019.2946980.

11. Chakraborty, D.; Elhegazy, H.; Elzarka, H.; Gutierrez, L. A Novel Construction Cost Prediction Model Using Hybrid Natural and Light Gradient Boosting. *Adv. Eng. Informatics* **2020**, *46*, doi:10.1016/j.aei.2020.101201.

12. Zheng, H.; Sherazi, S.W.A.; Lee, J.Y. A Stacking Ensemble Prediction Model for the Occurrences of Major Adverse Cardiovascular Events in Patients with Acute Coronary Syndrome on Imbalanced Data. *IEEE Access* **2021**, *9*, 113692–113704, doi:10.1109/ACCESS.2021.3099795.

13. Brieman, L. Stacked Regressions. *Mach. Learn.* **1996**, *24*, 49–64.

14. Chatzimparmpas, A.; Martins, R.M.; Kucher, K.; Kerren, A. StackGenVis: Alignment of Data, Algorithms, and Models for Stacking Ensemble Learning Using Performance Metrics. *IEEE Trans. Vis. Comput. Graph.* **2021**, *27*, 1547–1557, doi:10.1109/TVCG.2020.3030352.

15. Ganaie, M.A.; Hu, M.; Malik, A.K.; Tanveer, M.; Suganthan, P.N. Ensemble Deep Learning: A Review. *Eng. Appl. Artif. Intell.* **2022**, *115*, doi:10.1016/j.engappai.2022.105151.

16. Kansara, D.; Singh, R.; Sanghvi, D.; Kanani, P. Improving Accuracy of Real Estate Valuation Using Stacked Regression. *Int. J. Eng. Dev. Res.* **2018**, *6*, 571–577.

17. Neloy, A.A.; Sadman Haque, H.M.; Ul Islam, M.M. Ensemble Learning Based Rental Apartment Price Prediction Model by Categorical Features Factoring. *ACM Int. Conf. Proceeding Ser.* **2019**, *Part F148150*, 350–356, doi:10.1145/3318299.3318377.
