# Supplementary material for: Optimizing hypertension prediction using ensemble learning approaches
Source: PLoS One. 2024 Dec 23;19(12):e0315865. doi: 10.1371/journal.pone.0315865 (PMC11666061; doi:10.1371/journal.pone.0315865)
Supplement: S1 Table — (DOCX) [file pone.0315865.s002.docx]

Table S1: Demographic profiles of the respondents.

| **Risk Factor** |  | **Total** | **HTN** | **Non_HTN** | **P_value** |
| --- | --- | --- | --- | --- | --- |
|  | Cannot read and write | 139(22.71) | 33(23.7) | 106(76.3) | 0.595 |
|  | Read and write only | 98(16.01) | 16(16.3) | 82(83.7) |  |
| **Education** | Primary education (1–8) | 84(13.73) | 16(19.0) | 68(81.0) |  |
|  | Secondary education (9–12) | 108(17.65) | 22(20.4) | 86(79.6) |  |
|  | Diploma and above | 183(29.90) | 43(23.5) | 140(76.5) |  |
| **Physical activity** | Yes | 371(60.6) | 41(11.1) | 330(88.9) | <0.001 |
|  | No | 241(39.4) | 89(36.9) | 152(63.1) |  |
| **Age**,  mean (SD) |  | 47.56(13.40) | 54.66 (14.04) | 45.64(12.56) | <0.001 |
| **HHTN** | Yes | 84(13.7) | 45(53.6) | 39(46.4) | <0.001 |
|  | No | 528(86.3) | 85(16.1) | 443(83.9) |  |
| **HD** | Yes | 51(8.3) | 32(62.7) | 19(37.3) | <0.001 |
|  | No | 561(91.7) | 98(17.5) | 463(82.5) |  |
| **BMI** | Underweight | 10(1.63) | 2(10.0) | 8(80.0) | <0.001 |
|  | Normal | 366(59.80) | 49(13.4) | 317(86.6) |  |
|  | Overweight | 212(34.64) | 67(31.6) | 145(68.4) |  |
|  | Obese | 24(3.92) | 12(50.0) | 12(50.0) |  |
| ***Smoking*** | Yes | 74(12.1) | 29(29.2) | 45(60.8) | <0.001 |
|  | No | 538(87.9) | 101(18.8) | 437(81.7) |  |
| **Drinking** | Yes | 141(23.0) | 51(36.2) | 90(63.8) | <0.001 |
|  | No | 471(77.0) | 79(16.8) | 392(83.2) |  |
| **Salt** | Yes | 81(13.2) | 31(38.3) | 50(61.7) | <0.001 |
|  | No | 531(86.8) | 99(18.6) | 432(81.4) |  |
| **Fat** | Yes | 188(69.3) | 61(32.4) | 127(67.6) | <0.001 |
|  | No | 424(30.7) | 61(32.4) | 127(67.6) |  |
| **Model Transport** | On foot/pedal bicycle | 313(51.1) | 51(16.3) | 262(83.7) | 0.003 |
|  | Engine | 299(48.9) | 79(26.4) | 220(73.6) |  |
| **Vegetables** | Yes | 524(85.6) | 102(19.5) | 422(80.6) | 0.011 |
|  | No | 88(14.4) | 28(31.8) | 60(68.2) |  |
| **Weight,** mean (SD) |  | 66.59(8.769) | 73.07(9.462) | 64.84(7.698) | <0.001 |
| **Residence** | Urban | 408(66,.7) | 100(24.5) | 308(75.5) | 0.006 |
|  | Semi-urban | 204(33.3) | 30(14.7) | 174(85.3) |  |
| **Sex** | Male | 327(53.4) | 85(26.0) | 242(74.0) | 0.002 |
|  | Female | 285(46.6) | 45(15.8) | 240(84.2) |  |
| **MS** | Single | 29(4.47) | 4(13.8) | 25(86.2) | 0.795 |
|  | Married | 500(81.70) | 108(21.6) | 392(78.4) |  |
|  | Divorced | 24(3.92) | 5(20.8) | 19(79.2) |  |
|  | Widowed | 59(9.64) | 13(22.0) | 46(78.0) |  |
| **Religion** | Protestant | 337(55.07) | 62(18.4) | 275(81.6) | 0.147 |
|  | Orthodox | 181(29.58) | 50(27.6) | 131.(72.4) |  |
|  | Catholic | 38(6.21) | 6(15.8) | 32(84.2) |  |
|  | Muslim | 50(8.17) | 11(22.0) | 39(78.0) |  |
|  | Other | 6(0.98) | 1(16.7) | 5(83.3) |  |
| **Ethnicity** | Sidama | 222(36.27) | 43(19.4) | 179(80.6) | 0.285 |
|  | Walayita | 146(23.86) | 26(17.8) | 120(82.2) |  |
|  | Kembata | 105(17.16) | 24(22.9) | 81(77.1) |  |
|  | Guraga | 58(9.48) | 14(24.1) | 44(75.9) |  |
|  | Amahra | 44(7.19) | 15(34.1) | 29(65.9) |  |
|  | Oromo | 27(4.41) | 7(25.9) | 20(74.1) |  |
|  | Hadiya | 10(1.63) | 1(10.0) | 9(90.0) |  |
| **Occupation** | Employee | 199(32.52) | 32(16.1) | 167(83.9) | 0.033 |
|  | Daily-laborer | 53(8.66) | 9(17.0) | 44(83.0) |  |
|  | Merchant | 165(26.96) | 43(26.1) | 122(73.9) |  |
|  | Housewife | 118(18.28) | 22(18.6) | 96(81.4) |  |
|  | Retired | 59(9.64) | 20(33.9) | 39(66.1) |  |
|  | Others | 18(2.94) | 4(22.2) | 14(77.8) |  |
| **FM** | 1-3 | 138(22.55) | 22(15.9) | 116(84.1) | 0.062 |
|  | 4-6 | 281(45.92) | 57(20.3) | 224(79.7) |  |
|  | 7 or 7+ | 193(31.54) | 51(26.4) | 142(73.6) |  |
| **Income,** mean (SD) |  | 3169.69  (1999.468) | 3664.23  (2503.960) | 3036.31  (1820.149) | 0.001 |
| **Walking** | Yes | 471(77.0) | 63(13.4) | 408(86.6) | <0.001 |
|  | No | 141(23.0) | 67(47.5) | 74(52.5) |  |
| **Diabetes** | Yes | 561(91.67) | 98(17.5) | 463(82.5) | <0.001 |
|  | No | 51(8.33) | 32(62.7) | 19(37.3) |  |
| **Height,** mean (SD) |  | 165.20  (8.871) | 168.98  (8.062) | 164.18  (8.811) | <0.001 |
| **Kchat** | Yes | 526(85.9) | 107(20.3) | 419(79.7) | 0.200 |
|  | No | 86(14.1 | 23(26.7) | 63(73.3) |  |
| **Fruit** | Yes | 429(70.1) | 89(20.7) | 340(79.3) | 0.667 |
|  | No | 183(29.9) | 41(22.4) | 142(77.6) |  |
| **Wealth** | Poorest | 170(27.78) | 21(12.4) | 149(87.6) | 0.011 |
|  | Very poor | 103(16.83) | 24(23.3) | 79(76.7) |  |
|  | Poor | 117(19.12) | 29(24.8) | 88(75.2) |  |
|  | Less poor | 161(26.31) | 37(23.0) | 124(77.0) |  |
|  | Least poor | 61(9.97) | 19(31.1) | 42(68.9) |  |
