# Supplementary material for: Optimizing hypertension prediction using ensemble learning approaches
Source: PLoS One. 2024 Dec 23;19(12):e0315865. doi: 10.1371/journal.pone.0315865 (PMC11666061; doi:10.1371/journal.pone.0315865)
Supplement: S2 Table — (DOCX) [file pone.0315865.s003.docx]

Table S2: Risk factors for HTN identified using five feature selection techniques.

| **Boruta** | **Forward-Backward** | **Random Forest** | **Exhaustive Feature** | **Lasso Reg** | **Summary**  **(AՈB)** |
| --- | --- | --- | --- | --- | --- |
| Weight | Weight | Weight | Weight | Weight | Weight |
| Physicalinactivity | Physicalinactivity | Physicalinactivity | Physicalinactivity | Physicalinactivity | Physicalinactivity |
| Age | Age | Age | Age | Age | Age |
| History_HTN | History_HTN | History_HTN | History_HTN | History_HTN | History_HTN |
| History_DM | History_DM | History_DM | History_DM | History_DM | History_DM |
| BMI_cat | BMI_cat | BMI_cat | BMI_cat | BMI_cat | BMI_cat |
| Smoke | Smoke | Smoke | Smoke | Smoke | Smoke |
| Salt | Salt | Salt | Salt | Salt | Salt |
| Drink | Drink | Drink | Drink | Drink | Drink |
| Fat | Fat | Fat | Fat | Fat | Fat |
| Model_transport | Model_transport | Model_transport | Model_transport | Model_transport | Model_transport |
| Vegetables | Vegetables | Vegetables | Vegetables | Vegetables | Vegetables |
| Education_status | Education_status | Education_status | Education_status | Education_status | Education_status |
| Height | kchat | Height | kchat | Height | Total 13 Feature |
| Occupation |  | Occupation |  | Occupation |  |
| Average monthly Income |  | Average monthly Income |  | Ethnicity |  |
| Wealth indes_cat |  | Wealth indes_cat |  | PermanentResidence |  |
| Family size |  | Family size |  | marital_new |  |
| Sex |  | Religious affiliation |  | Sex |  |
|  |  | Ethnicity |  | kchat |  |
