# Supplementary material for: Optimizing hypertension prediction using ensemble learning approaches
Source: PLoS One. 2024 Dec 23;19(12):e0315865. doi: 10.1371/journal.pone.0315865 (PMC11666061; doi:10.1371/journal.pone.0315865)
Supplement: S3 Table — (DOCX) [file pone.0315865.s004.docx]

**Table S3:** Performance evaluation of prediction models on imbalanced data.

| **Data** | **Models** | **Accuracy** | **Precision** | **Recall** | **F1-Score** | **AUC** |
| --- | --- | --- | --- | --- | --- | --- |
| Imbalance Data | LightGBM | 73.07 | 69.23 | 75.0 | 78.94 | 0.807 |
|  | LR | 79.48 | 76.92 | 81.08 | 78.94 | 0.858 |
|  | ANN | 75.64 | 71.79 | 79.49 | 75.44 | 0.799 |
|  | RF | 73.08 | 64.52 | 78.72 | 70.91 | 84.01 |
|  | XGB | 66.67 | 64.10 | 67.56 | 65.78 | 0.667 |
