# Supplementary material for: Optimizing hypertension prediction using ensemble learning approaches
Source: PLoS One. 2024 Dec 23;19(12):e0315865. doi: 10.1371/journal.pone.0315865 (PMC11666061; doi:10.1371/journal.pone.0315865)
Supplement: Attachment — Submitted filename: Response to Reviewers.docx [file pone.0315865.s006.docx]

**Response to Reviewer Comments**

**Reviewer #1:**

**General Comment:** This study focuses on predicting hypertension using the improved machine learning approach, demonstrating impressive accuracy and superiority over other known machine learning methods. This topic is crucial due to the high prevalence of hypertension, a chronic disease often unrecognized for years in some patients, leading to a high rate of complications. Early identification of risk factors and timely intervention are essential for effective prevention. The manuscript is clearly written, but several significant limitations must be addressed before it is ready for publication.

**Response:** Thank you so much for your important comments that help us to improve the quality of the manuscript.

**Comment 1:** Study Design and Data Limitations:

The data utilized in this study were obtained through a cross-sectional design, which only reveals a temporal association between the examined factors and hypertension. Due to this study design:

• The data cannot establish a causal relationship between any examined factor and hypertension. Based solely on the presented data, the authors cannot state whether some of these factors contribute to the development of hypertension (as mentioned in line 294), if they are a consequence of it, or if their association with hypertension is purely coincidental. However, previous studies have established causal links between the described factors and hypertension. This needs to be mentioned in the discussion section as one of the important limitations of the study.

**Response:** Thank you for your valuable comment. We revised this statement in the revised manuscript. Please see lines 334-336 and discussion section for details.

• The data cannot be used to predict whether an individual will develop hypertension in the future. Traditional methods for risk prediction, already in use in the everyday clinical practice, are able to predict the future risk over a span of 1 to 4 years. The authors need to please address this in the discussion section.

**Response:** Thank you for your comment. We have revised it in the revised manuscript accordingly. Please see the discussion section.

• Additionally, the authors need to discuss whether the other machine learning methods mentioned in Table 3 are based on cross-sectional data, or if they predict current or future risk.

**Response:** Thank you for your valuable comment. We have revised it accordingly in the discussion section. Please see the Table 3 and lines 436, 437.

**Comment 2:** Comparison with Classical Risk Prediction Tools:

The discussion section should highlight a comparison of the presented results with classical risk prediction tools currently used in clinical practice, not just other machine learning methods (e.g., the hypertension risk calculator from the Framingham study). How does this method differ from or improve upon these existing tools?

See references:

<https://pubmed.ncbi.nlm.nih.gov/18195335/>
<https://www.ncbi.nlm.nih.gov/pmc/articles/PMC8110170/>

**Response:** Thank you for your valuable suggestion. We have revised the manuscript accordingly. Please see lines 437-448 for details.

**Comment 3:** Furthermore, please discuss the rationale behind calculating the current risk of hypertension. Wouldn't it be simpler and more straightforward to just measure the patient's blood pressure? Predicting future risk seems more valuable, as it could facilitate early identification of at-risk individuals and allow for timely interventions to prevent or mitigate the development of hypertension (before we can actually detect hypertension by measurement).

**Response:** Thank you for your valuable suggestion. We have revised it in the revised manuscript. Please see lines 395-401.

**Comment 4:** Inclusion Criteria and Population Specificity:

Please clarify the inclusion criteria used for recruiting participants in this study. Were participants randomly selected from the general population, or were there specific selection criteria? This information is crucial for readers to understand the applicability of this predictive approach to different populations. For instance, were the participants exclusively adults (older than a certain age), were female participants exclusively non-pregnant, and were elderly individuals (65 and older) included, given the increased risk of hypertension with age?

**Response:** Thank you for your comment. We have clarified it in the revised manuscript. Please see lines 99, 100.

**Reviewer #2**

The paper titled "Optimizing Hypertension Prediction Using Ensemble Learning Approaches" addresses an important issue in healthcare: the prediction of hypertension, a leading cause of morbidity and mortality worldwide. The study employs ensemble learning methods, specifically a stacking model, to enhance predictive accuracy compared to traditional single-model approaches. The authors utilize a dataset comprising 612 participants from Ethiopia and report significant improvements in predictive performance. The subject matter of this study is highly relevant, particularly given the global burden of hypertension and the increasing focus on personalized medicine. The authors have chosen an appropriate methodological approach by leveraging ensemble learning, which is well-suited to the complexities of hypertension prediction. Additionally, the use of SHapley Additive Explanations (SHAP) for interpreting the model's predictions is a commendable choice, as it adds a layer of interpretability, making the results more actionable in a clinical setting. However, multiple aspects of the paper require further attention.

**Response:** Thank you so much for your important comments that help us to improve the quality of the manuscript.

Firstly, while the use of ensemble learning is methodologically sound, the manuscript does not introduce a significant theoretical or methodological innovation. Ensemble learning techniques, particularly stacking, are well established in the field of machine learning. For the manuscript to be considered for publication in a journal, it would benefit from demonstrating a novel algorithm, a unique application of existing methods, or a substantial improvement over current benchmark. As it stands, the contribution of this work appears to be incremental rather than transformative. A more in-depth comparative analysis and a clearer articulation of what differentiates this approach from existing work could strengthen the paper.

**Response:** Thank you for your valuable comment. We have revised the manuscript accordingly. Please see the lines 82-94.

Another concern is the data limitations. The study is based on a dataset of 612 participants, which is relatively small for machine learning applications. This limitation raises questions about the generalizability of the findings. Although the authors have employed data balancing techniques such as ADASYN, the small sample size could still lead to overfitting, thus limiting the external validity of the results. Expanding the dataset or providing a stronger justification for its representativeness would greatly enhance the credibility of the study.

**Response:** Thank you for your insightful comment regarding the data limitations. We acknowledge that the sample size of 612 participants may be relatively small for machine learning applications, which could raise questions about the generalizability of our findings. Our dataset was taken from secondary data collected in a published study, and although it may appear limited, we employed the ADASYN technique to address class imbalance and help mitigate potential overfitting. Moving forward, we aim to expand the dataset and provide a stronger justification for its representativeness to enhance the credibility of our study. Please see lines 263-268 for details.

The manuscript also faces challenges in language and clarity. There are numerous grammatical errors and instances of unusual phrasing that detract from the readability and overall professionalism of the paper. Additionally, inconsistencies in terminology, such as the incorrect spelling of "Shapley Additive Explanations," further undermine the manuscript’s presentation. A thorough revision for language clarity and grammatical accuracy is strongly recommended.

**Response:** Thank you for your suggestion. We have conducted a thorough revision of the manuscript to enhance language clarity, ensure grammatical accuracy, and maintain consistency in terminology throughout the paper.

While the statistical methods employed are appropriate, they are standard. The paper would benefit from a more detailed discussion of why specific methods were chosen and how they contribute to the study’s goals. The SHAP analysis, while useful, could be explored in greater depth. The authors should consider elaborating on the clinical implications of their findings and how these results compare with established risk factors for hypertension.

**Response:** Thank you for comment. We have revised the manuscript accordingly. Please see lines 133-154, 436-448 for details.

While the data were sourced from a prior study, it would be good to include a brief statement regarding the ethical approval for this secondary analysis, particularly given the sensitive nature of health-related data.

**Response:** Thank you for your suggestion. We have included a statement regarding ethical approval in the revised manuscript to enhance clarity and transparency. Please see the ethical approval section for details.

This paper addresses a well-known issue in healthcare and offers a solid application of ensemble learning for hypertension prediction, but it requires significant revisions to meet the standards of a journal. Enhancing the novelty of the contribution, addressing the limitations related to data size and generalizability, and refining the language and structure of the manuscript is crucial.

**Response:** Thank you for your feedback. We appreciate your recognition of the issue addressed and the application of ensemble learning in hypertension prediction. We have revised the manuscript accordingly.

**Reviewer #3**

The manuscript addresses an important area in healthcare analytics by using ensemble learning approaches to predict hypertension. Proposed application of multiple machine learning models, combined with feature selection and hyperparameter tuning, can further contribute to this research field. However, there are several areas where this research falls short, particularly concerning data employed, methodology description, presentation of results, clarity and novelty of methods applied.

**Response:** Thank you so much for your important comments that help us to improve the quality of the manuscript.

**Comment 1**: The manuscript lacks discussion of the limitations associated with the dataset, particularly sample size (only 612 instances), potential biases in data (21%HTN vs 79% no HTN). The quality of the dataset raises some concerns, which could impact the overall integrity of the study's findings.

**Response:** Thank you for your valuable comment. We acknowledge the limitations regarding the sample size of 612 instances and the imbalance between HTN (21%) and non-HTN (79%) cases. We have addressed these in the limitations section.

**Comment 2:** The study also lacks a strong description of how the data was handled (e.g. data preparation, cleaning, outliers etc.

**Response:** Response: Thank you for the comment. We have clarified the data handling process in the revised manuscript. Please see lines 110-114.

**Comment 3:** This study utilized secondary data on HTN collected from Paulose et al., (2022), where they investigated the prevalence and associated factors of HTN among the population of Hawassa city in Ethiopia. The reported performance metrics are notably high, especially given the small sample size. It would be beneficial to (if possible) test the stacked model on an independent dataset. The high and well-balanced metrics of the proposed stacked model using ADASYN are promising, but due to the relatively small dataset, may not be representable, especially when these inconsistencies are present. Also, further discussion of how the proposed method yielded such results is needed.

**Response:** Thank you for your insightful comment. We acknowledge the concern regarding the relatively small dataset and the potential implications for the representativeness of our findings. While the high performance metrics of the stacked model using ADASYN are promising, we recognize that further validation is needed. Testing the model on an independent dataset would strengthen the generalizability of the results. Unfortunately, access to additional data was not available at this stage, but future work will focus on expanding the dataset or applying the model to other populations. We have also expanded the discussion to provide a more detailed explanation of how the proposed method, including ADASYN, contributed to the performance metrics observed in this study.

**Comment 4**: The manuscript’s methodology description contains high level technical detail, particularly in the description of the machine learning algorithms. These details can be important, but as there are no implications or discussions on potential modifications to the models, means that these descriptions may be excessive, as they primarily serve to outline the models rather than contribute to further experimentation or development. Consider simplifying or moving these discussions to the appendix.

**Response:** Thank you for your valuable suggestion. We have moved this discussion to the appendix in the revised manuscript.

**Comment 5:** The description of the model in focus (stacked model) should be head lighted by rigorous description and discussion. It would be beneficial to further discuss the rationale behind using Logistic Regression as the level 1 learner (does this choice assume linearity in the dataset? What happens with complex relationships?) Furthermore, why has LR been used as both level 0 and level 1 learner? What about posing other models as level 1 learners? What advantages or improvements might other models offer in terms of capturing complex relationships or enhancing the overall predictive performance?

**Response:** Thank you for your valuable comment. We have revised the manuscript and included a more rigorous description and discussion of the stacked model, specifically addressing the choice of LR as the level 1 learner. Please see lines 146-156.

**Comment 6**: The use of SHAP values is the strength of the manuscript, but the discussion of how these values contribute to the interpretability of the models can be expanded. Specifically, the authors should provide more insight into how the identified risk factors could be used in clinical practice to guide decision-making. From a practical viewpoint, this would enhance the relevance of the study. Consider including an ethics statement in the manuscript, as the data used are clinical in nature.

**Response:** Thank you for your valuable comment. We acknowledge that while the use of SHAP values is indeed strength of our manuscript, the discussion surrounding their contribution to model interpretability can be expanded. We have revised the manuscript accordingly. Additionally, we have included an ethics statement in the manuscript, addressing the clinical nature of the data used in our analysis.
