# Supplementary material for: Optimizing hypertension prediction using ensemble learning approaches
Source: PLoS One. 2024 Dec 23;19(12):e0315865. doi: 10.1371/journal.pone.0315865 (PMC11666061; doi:10.1371/journal.pone.0315865)
Supplement: Attachment — Submitted filename: Response to Reviewers.docx [file pone.0315865.s007.docx]

**Response to Academic Editor/Reviewer Comments**

**Academic Editor:**

**Comment 1**: Please note the suggestion to further research and or consult a medical doctor to address gaps in basic knowledge on hypertension and ensure accuracy, as well as to refine certain wording and interpretations, particularly when comparing with established tools like the Framingham risk score. This is critical due to the interdisciplinary nature of the journal and your manuscript.

**Response:** Thank you for your valuable comment. We consulted a medical doctor and, based on their suggestions, revised the interpretations particularly in comparisons with established tools like the Framingham risk score (see introduction section and lines 336-341).

**Comment 2:** If possible consider discussiing other risk calculators (e.g., STRONG HEART study).

**Response:** Thank you for your suggestion. We have incorporated a discussion on the STRONG HEART study, another risk calculator, to provide a broader context and enhance the manuscripts comprehensive (see lines 341-351).

**Comment 3:** Please have the text checked for for grammar and style improvements.

**Response:** Thank you for the suggestion. We have thoroughly reviewed the text to address grammar and style issues, ensuring clarity and readability throughout the manuscript.

**Reviewer #1:**

**Comment:** I appreciate the authors' efforts to incorporate the required changes; however, some revisions appear to be somewhat superficial and require further refinement. I recommend that they consult a medical doctor to review the text before resubmission, as the current draft contains several misinterpretations that indicate a gap in basic knowledge on the subject of hypertension. Addressing these issues would help to better showcase the hard work and dedication the authors have invested in this study.

Lines 445 to 456 (in the file with the tracked changes): I recommend a more careful choice of wording in this section. When comparing the proposed method with the established Framingham risk-prediction tool commonly used in clinical practice, it is important to note that the Framingham tool has been validated in a study of over 1,000 participants, has been in use for many years, and has demonstrated lasting reliability. Moreover, this classical hypertension risk score can predict hypertension up to four years before onset, which offers a distinct advantage over the approach in the current study. The present study, in contrast, is based on a smaller sample size and is focused primarily on predicting current hypertension status.
It’s worth acknowledging, however, that this classic tool from Frminham stuy may lack generalizability to ethnically diverse populations, as it was developed in a study focused on people of European descent. The newly proposed method could serve as a complementary approach to existing risk scores, as it identifies individuals currently suffering from hypertension who might otherwise be missed without a recent blood pressure check. The study’s results are also based on a population of different ethnicity, which may enhance its relevance across broader demographic groups.

The authors should consider discussing other hypertension risk calculators, such as the STRONG HEART study, which is particularly suited to Native American populations.

It would also be valuable to comment on whether the risk factors identified in these other studies are consistent with or differ from those proposed in this study.

Additionally, it would be beneficial for a native English speaker to review the text to improve grammar and style. For example, tin the introduction section, the term “cardiovascular disease” is missing from the second sentence, and the third sentence should use “cause” instead of “causes.”

**Response:** We sincerely thank the reviewer for their valuable feedback to improve the quality of the manuscript. To address the concerns, we consulted a medical professional specializing in hypertension to ensure the accuracy of our interpretations (please see the introduction and discussion section). The manuscript now better highlights the strengths of the Framingham tool, including its long-term validation and predictive capabilities, while positioning our method as a complementary approach that identifies current hypertension cases, particularly in ethnically diverse populations (see lines 336-341). We also incorporated a discussion on the STRONG HEART study and compared risk factors identified in our study with those from other research (please see lines 341-351). Also, we have expanded our analysis to comment on whether the risk factors identified in other studies align with or differ from those identified in our study (please see lines 353-377). Additionally, we thoroughly reviewed the text to improve clarity, grammar, and style, incorporating specific corrections to the introduction as suggested. These revisions aim to address all concerns and enhance the overall quality of the manuscript.
